# Neuroplasticity in Spinal Cord Injury: A Scoping Review of Characterized Mechanisms

**DOI:** 10.3390/biomedicines14091963

**Published:** 2026-08-31

**Authors:** Luong Tuan Khanh, Le Thi Ha, Tran Thai Hung, Doan Thanh Nhan, Nguyen Thi Kim Lien, Bui Thi Hoai Thu, Duong The Vinh, Tran Thanh Tinh, Tran Huu Dat

**Affiliations:** 1 Center of Rehabilitation, Bach Mai Hospital, Hanoi 100000, Vietnam; ltkrehab.bm@gmail.com (L.T.K.); mi.hoaithu@gmail.com (B.T.H.T.); 2 Department of Rehabilitation, Haiduong Medical Technical University, Haiduong 030000, Vietnam; 3 Department of Rehabilitation, Vinmec Smart City Hospital, Vinmec International Hospital, Hanoi 120000, Vietnam; v.hungtt44@vinmec.com; 4 Department of Rehabilitation, Vinmec Da Nang Hospital, Vinmec International Hospital, Da Nang 550000, Vietnam; drchannhan@gmail.com; 5 Department of Rehabilitation, Hanoi Medical University, Hanoi 100000, Vietnam; lienrehab@yahoo.com; 6 Department of Rehabilitation, Vietduc University Hospital, Hanoi 100000, Vietnam; 7 Department of Rehabilitation, Vinmec Times City International Hospital, Vinmec International Hospital, Hanoi 100000, Vietnam; v.vinhdt@vinmec.com (D.T.V.); v.tinhtt6@vinmec.com (T.T.T.); 8 College of Health Sciences, VinUniversity, Hanoi 124000, Vietnam

**Keywords:** spinal cord injury, neuroplasticity, cortical reorganization, rehabilitation

## Abstract

**Background/Objectives**: Neuroplasticity is crucial for functional adaptation after spinal cord injury (SCI). Both adaptive/maladaptive changes occur across the cortical/subcortical/spinal levels, influencing recovery, compensation, and long-term complications. However, the contributions of these mechanisms remain unclear. This study investigated the current evidence on neuroplastic mechanisms following SCI, identifying knowledge gaps relevant to rehabilitation/functional recovery. **Methods**: This scoping review searched the PubMed, Scopus, and Cochrane databases from inception to 14 November 2025. Neuroimaging/electrophysiological methods used to investigate neuroplastic changes in human SCI were assessed. Data on study design, participant characteristics, injury features, methodological approaches, and reported neuroplastic mechanisms were extracted. Findings were categorized into predefined domains (axonal regeneration, synaptic plasticity, cortical reorganization, propriospinal pathway reorganization, neurochemical and molecular changes, activity-dependent plasticity, maladaptive plasticity and other mechanisms). **Results**: Across 37 studies, cortical reorganization was most commonly reported (*n* = 19), followed by activity-dependent plasticity (*n* = 14), and synaptic plasticity (*n* = 12). Propriospinal pathway reorganization and maladaptive plasticity were reported in six and four studies, respectively. Evidence on axonal regeneration (*n* = 1) and neurochemical/molecular mechanisms (*n* = 2) were limited. Neuroimaging/electrophysiological findings demonstrated dynamic cortical reorganization, altered sensorimotor network connectivity, and progressive structural changes in motor pathways. Activity-dependent interventions, including task-specific training, motor imagery, and neuromodulation, would appear to promote adaptive plasticity, while maladaptive changes are associated with neuropathic pain, abnormal excitability, and compensatory overactivation. **Conclusions**: Neuroplasticity after SCI involves multiple interactions with cortical/spinal mechanisms. Cortical reorganization and activity-dependent plasticity were the most frequently investigated neuroplasticity domains identified in the included studies, while axonal regeneration and molecular mechanisms remain underexplored in humans. Future multimodal longitudinal studies are needed to clarify these relationships and optimize rehabilitation strategies.

## 1. Introduction

Spinal cord injury (SCI) is a severe neurological condition characterized by the partial or complete disruption of motor, sensory, and autonomic pathways below the level of lesion. In addition to immediate loss of function, individuals with SCI commonly experience long-term complications, including impaired mobility, autonomic dysfunction, and chronic pain, all of which significantly reduce quality of life [1,2]. Despite advances in acute management and rehabilitation, functional recovery after SCI remains limited, highlighting the need for a better understanding of the mechanisms underlying neurological adaptation and recovery.

Neuroplasticity, defined as the structural/functional reorganization capacity of the central nervous system in response to an injury or experience, has emerged as a key mechanism in the response to SCI [3]. Following injury, the brain and spinal cord undergo a range of adaptive and maladaptive changes, including cortical reorganization, alterations in synaptic efficacy, and modifications in large-scale neural networks [4,5,6]. These changes are driven by the loss of afferent inputs and efferent outputs, as well as by the compensatory use of preserved neural pathways, and may evolve over time from the acute to chronic injury stages.

Accumulating evidence from multimodal neuroimaging and electrophysiological studies has demonstrated that neuroplasticity following SCI occurs at multiple levels [5,6,7,8]. For example, the reorganization of sensorimotor representations at the cortical level has been widely reported, commonly involving shifts in somatotopic maps and changes in cortical excitability [6,7]. At the network level, altered functional connectivity within and between sensorimotor and associative brain regions reflects both compensatory recruitment and efficiency reorganization [9]. In parallel, structural imaging studies have shown progressive spinal cord atrophy and reductions in cortical white and gray matter volume within descending motor pathways [10], indicating that plasticity and degeneration may coexist. Furthermore, activity-dependent mechanisms, including long-term potentiation–like plasticity, play critical roles in shaping the functional outcomes and responsiveness to rehabilitation [11].

Neuroplasticity following SCI may have both adaptive and maladaptive effects. While adaptive plasticity may support functional recovery and compensation, maladaptive changes have been implicated in persistent complications such as neuropathic pain and spasticity [12,13,14]. The balance between these processes is influenced by multiple factors including injury severity, time since injury, and rehabilitation interventions [7]. Given the growing body of evidence and the heterogeneity of findings across studies, a comprehensive synthesis of current knowledge is required. This article aimed to review and integrate existing evidence on neuroplasticity after SCI, focusing on cortical reorganization, network-level adaptations, and underlying mechanisms, as well as their clinical implications for rehabilitation and recovery.

## 2. Materials and Methods

This scoping review was conducted in accordance with the Preferred Reporting Items for Systematic Reviews and Meta-Analyses extension for Scoping Reviews (PRISMA-ScR) guideline [15]. Completed PRISMA for Abstracts and PRISMA-ScR checklists are provided in Supplementary Tables S1 and S2, respectively. Protocol registration was not performed because PROSPERO currently does not accept scoping review protocols.

### 2.1. Search Strategy

The primary review question was focused on the neuroplastic changes and mechanisms described following spinal cord injury. A comprehensive literature search was conducted using the PubMed, Scopus, and Cochrane Library electronic databases for all relevant articles published up to 14 November 2025. Keywords and Medical Subject Headings (MeSH) related to SCI and neuroplasticity were used in various combinations, including “spinal cord injury”, “SCI”, “neuroplasticity”, and “neural reorganization”. Boolean operators (AND and OR) were used to optimize the search strategy for each database. Please see the Appendixes A–C for the detail.

### 2.2. Eligibility Criteria

The inclusion criteria were:

1. Investigated neuroplastic changes following spinal cord injury in human participants.
2. Employed neuroimaging techniques (e.g., functional Magnetic Resonance Imaging [fMRI], Diffusion Tensor Imaging [DTI], Positron Emission Tomography [PET]) or electrophysiological techniques (e.g., Transcranial Magnetic Stimulation [TMS]).
3. Reported outcomes related to cortical, subcortical, or network-level reorganization.

The exclusion criteria were:

1. Studies conducted exclusively in animal models;
2. Studies not directly assessing neuroplasticity or brain/spinal reorganization;
3. Conference abstracts, editorials, or non-peer-reviewed articles;
4. Articles not available in English.

### 2.3. Study Selection

All identified records were imported into a reference management website (https://new.rayyan.ai/ (accessed on 27 February 2026)), and duplicates were removed. Subsequently, two authors independently screened the titles and abstracts to identify any potentially relevant studies by two authors. Full-text articles were assessed for eligibility based on the predefined inclusion and exclusion criteria. Discrepancies were resolved through discussions.

### 2.4. Data Extraction and Synthesis

Relevant data were extracted from each included study, including the study design, sample characteristics, injury features (level, severity, and duration), methodological approach (e.g., neuroimaging or electrophysiological techniques), and key findings related to neuroplasticity.

Findings were categorized according to predefined neuroplasticity mechanisms, including axonal regeneration, synaptic plasticity, cortical reorganization, propriospinal pathway reorganization, neurochemical and molecular changes, activity-dependent plasticity, and maladaptive plasticity. Classification was based on the predominant mechanisms and primary focus of each study, following discussion and consensus among the authors. Because these neuroplasticity concepts are not entirely distinct and may overlap, some studies could be relevant to more than one category. Studies that did not clearly fit these categories were grouped according to other mechanisms.

Narrative synthesis was conducted, and the results were organized according to these mechanistic domains to provide a comprehensive overview of the current evidence of neuroplasticity following spinal cord injury.

## 3. Results

**Figure 1:**
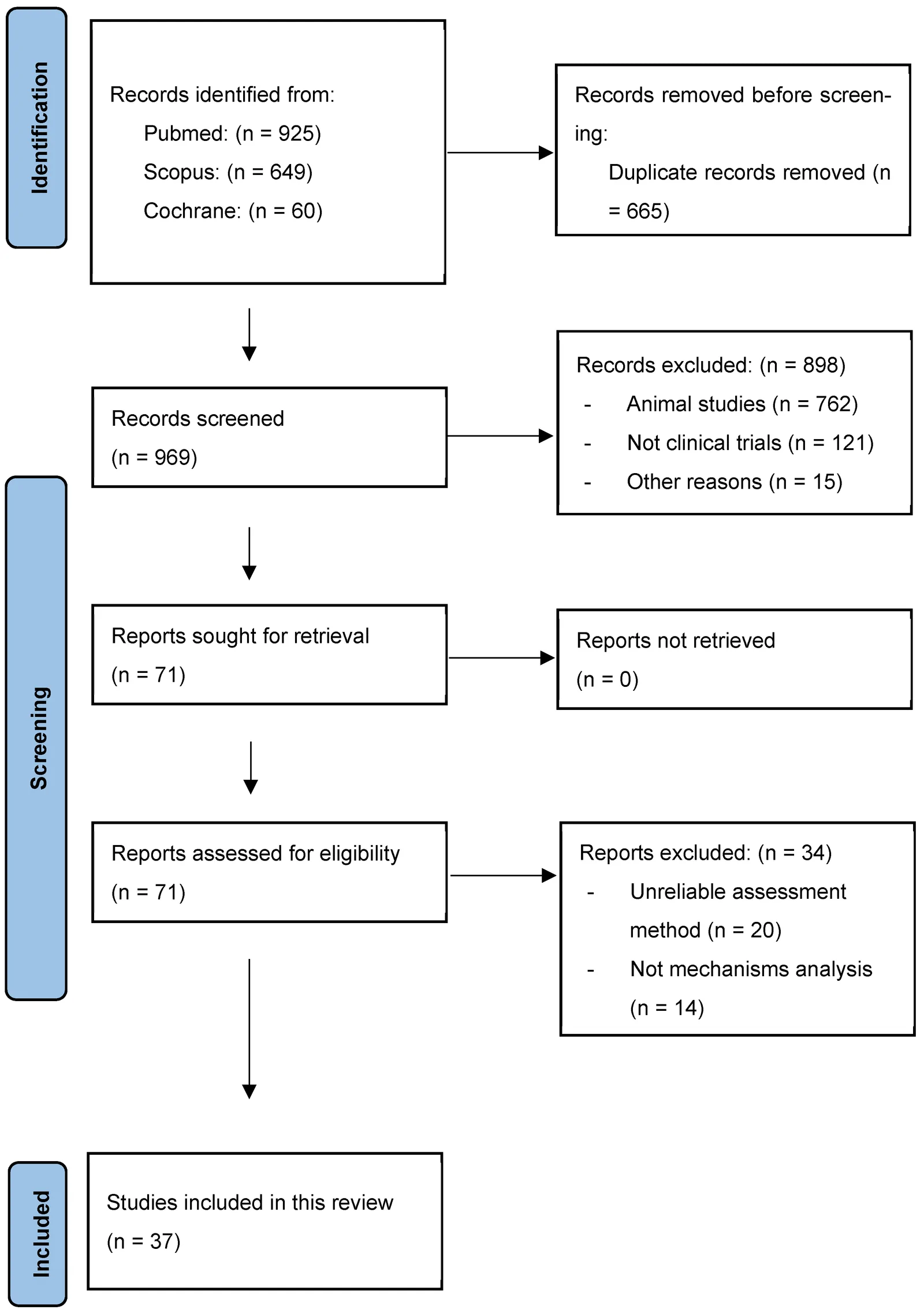
PRISMA flow diagram.

After screening 969 records and assessing 71 full-text articles, and following exclusion based on predefined criteria, 37 studies were included in the final review (Figure 1).

Among the included studies, 976 individuals with SCI were reported with a wide range of injury levels, severities, and durations. Cortical reorganization was the most commonly investigated mechanism (*n* = 19), followed by activity-dependent plasticity (*n* = 14) and synaptic plasticity (*n* = 12). Reorganization of the propriospinal pathway was reported in a moderate number of studies (*n* = 6), whereas maladaptive plasticity was less commonly examined (*n* = 4). Only a small number of studies addressed axonal regeneration (*n* = 1) or neurochemical and molecular changes (*n* = 2), with other mechanisms identified in two studies (Table 1).

Most studies employed cross-sectional or case–control designs, while a smaller number used longitudinal, interventional, or randomized controlled trial designs. Sample sizes varied considerably, ranging from single case reports to large cohort studies. Neuroimaging and electrophysiological techniques were the primary methodological approaches across the included studies. fMRI, TMS, and EMG were the most commonly used methods, while structural MRI and DTI were also frequently applied. In contrast, EEG, MEG (Magnetoencephalography), and PET were less frequently reported.

## 4. Discussion

This scoping review summarizes the current evidence on neuroplasticity following SCI and organizes the findings into several thematic domains, including cortical reorganization, activity-dependent plasticity, synaptic plasticity, propriospinal pathway reorganization, axonal regeneration, neurochemical and molecular changes, and maladaptive plasticity. Among these, cortical reorganization has emerged as the most frequently investigated mechanism, followed by activity-dependent synaptic plasticity. These findings highlight that neuroplasticity after SCI is a multifaceted process involving both adaptive and maladaptive changes at the cortical, subcortical, and spinal levels.

### 4.1. Activity-Dependent Plasticity

Activity-dependent plasticity, the foundation of modern rehabilitation methods, is based on the principle that repeated performance of a motor task or exposure to a stimulus can reshape the structure and function of neural networks [53]. Evidence suggests that the brain and spinal cord can adapt to specific training and neural stimulation. Specifically, some studies have indicated that intensive training promotes specific changes in spinal reflex excitability. For example, endurance training via treadmill walking enhances inhibition of the cutaneomuscular reflex (CMR) in the soleus muscle, a factor correlated by improved walking speed and distance [54]. Regarding upper limb and hand function recovery, the volume of the primary motor cortex gradually increases (up to 74%), while activation in compensatory regions decreased [22]. The combination of bimanual mass practice and somatosensory stimulation significantly increased the area and volume of the motor cortex map corresponding to the biceps muscle. The expansion and forward shift in the center of gravity (COG) of the motor cortex map are accompanied by improvements in motor skill performance of both hands [21]. In addition, maintaining regular muscle activity through stimulation or exercise reversed H-reflex depression and facilitated the rehabilitation process [32,55]. In a study on motor imagery (MI), after five weeks of MI training for tenodesis grasp, patients showed reduced compensatory recruitment, with brain maps becoming more specific and starting to resemble those of healthy individuals. The number of cortical activation sources (measured by MEG) directly predicted a reduction in the variability of actual movements, thereby resulting in smoother movements. This demonstrates that the cortical network controlling imagined movements is reorganized in parallel and linked to the recovery of actual motor function [36,37].

A combination of training and neurostimulation methods may enhance activity-dependent plasticity. One clinical trial in patients combined exercise with paired corticospinal motor neuron stimulation (PCMS). PCMS (with or without exercise) increased the maximum voluntary contraction (MVC) and motor-evoked potentials (MEPs) by 40–50%. PCMS combined with training helped to maintain these functional benefits for up to 6 months [48]. Transcutaneous electrical stimulation of the spinal cord, combined with robot-assisted gait training, further reduced the excitability of the flexor reflex, and increased the output of motor neurons across multiple spinal segments [46].

Flexibility is also influenced by the nature of the exercise (training-specific). In one study comparing two forms of walking training in patients with incomplete SCI, only endurance training increased the inhibitory intensity of the cutaneous-muscular reflex (CMR) in the soleus muscle during the gait cycle, whereas precision training did not produce this effect. More importantly, although the intensity of muscle clonus may not decrease uniformly across all patients, in individuals experiencing a reduction in clonus, this reduction correlates with improvements in the actual walking speed and distance [54]. Overall, these results demonstrate that the spinal network learns to enhance inhibition through continuous repetition, optimizing firing patterns to facilitate motor recovery.

### 4.2. Cortical Reorganization

Overall, we identified 18 studies that demonstrated cortical reorganization in individuals with SCI, making it the most widely reported neuroplasticity mechanism in this population.

Functional MRI evidence from patients with cervical spinal cord compression has demonstrated that cortical reorganization involves differential changes in the motor and sensory cortices related to treatment and recovery. Prior to decompression, patients exhibit increased activation in the primary motor cortex (precentral gyrus), concurrent with reduced activation in the primary somatosensory cortex (postcentral gyrus), reflecting compensatory motor recruitment and impaired sensory input [28]. Following surgical decompression, there was a significant expansion of activation in both the motor and sensory cortices, accompanied by clinical improvement, suggesting partial restoration and adaptive cortical recruitment [28]. These findings indicate that cortical reorganization after spinal cord injury is dynamic and region-specific, with increased motor cortex engagement and gradual normalization of sensory cortex activity that parallels functional recovery. In another study using resting-state fMRI with graph-theoretical analysis, Kaushal et al. [42] demonstrated that spinal cord injury (SCI) induces the reorganization of large-scale brain networks, particularly within the sensorimotor–cerebellar circuitry. While the overall whole-brain connectivity is reduced, reflecting disrupted afferent and efferent signaling, a specific subnetwork linking the sensorimotor cortex and cerebellum shows increased functional connectivity [42]. This enhanced coupling, particularly involving the paracentral lobule, indicates compensatory synchronization and recruitment of the cortico-cerebellar pathways to support motor control in the absence of normal spinal input. These findings highlight that cortical–cerebellar network reorganization is a key adaptive mechanism within broader network-level plasticity following SCI.

Findings from multimodal neuroimaging and electrophysiological studies have suggested that neuroplasticity following SCI involves enhanced recruitment and strengthening of sensorimotor networks, particularly supporting preserved and compensatory motor functions. Studies have further shown enhanced connectivity between the motor, sensory, and associative regions, such as increased coupling between the primary motor cortex and parietal areas, as well as expansion and redistribution of the somatosensory representations, supporting improved control of intact limbs and compensatory movements [36,39,41,50]. In addition, activity-dependent interventions (e.g., motor imagery and task-specific training) can modulate these networks, reducing maladaptive overactivation, and promoting more efficient and normalized activation patterns [21]. Longitudinal and outcome-based studies have further demonstrated that increased functional connectivity within motor networks is associated with better recovery, whereas reduced connectivity correlates with poorer outcomes [36,39,41,50]. Collectively, these findings indicate that increased sensorimotor network engagement is a key adaptive mechanism underlying functional compensation and recovery after SCI.

Structural MRI and diffusion imaging studies have suggested that neuroplasticity following SCI is accompanied by progressive neurodegenerative changes that affect both the cortical regions and descending motor pathways. Reduced gray matter volume in the primary motor and somatosensory cortices, along with decreased fractional anisotropy and increased diffusivity in corticospinal and corticopontine tracts, all reflect axonal loss, demyelination, and structural disintegration of the neural tissue [18,25,31]. These changes are consistent with retrograde degeneration mechanisms, including apoptosis of the corticospinal neurons, as demonstrated in animal models and supported by imaging correlates in humans [18]. Additionally, degeneration extends beyond the primary motor areas, involving the cerebellar and prefrontal regions, indicating widespread secondary neurodegeneration following the loss of afferent and efferent signaling. Importantly, the degree of structural atrophy may be modulated by concurrent cortical reorganization, suggesting an interaction between degenerative and plastic processes [18,25,31]. Collectively, these findings indicate that neurodegeneration, likely partly driven by apoptosis and disconnection, is a key component of maladaptive neuroplasticity following SCI.

Evidence from functional and structural neuroimaging studies has indicated that neuropathic pain following spinal cord injury (SCI) is closely linked to maladaptive cortical reorganization, particularly within the primary somatosensory cortex (S1) and broader pain-related networks. Specifically, shifts in S1 somatotopic representations, such as the displacement of upper limb representations towards deafferented lower limb areas, are strongly correlated with pain intensity, indicating that greater cortical reorganization is associated with more severe neuropathic pain [26]. Beyond S1, diffusion imaging studies have revealed structural alterations in multiple supraspinal regions involved in pain processing and modulation, including the thalamus, insula, prefrontal cortex, amygdala, and posterior parietal cortex, with the magnitude of these changes correlating with pain severity [30]. Overall, these findings support the concept that neuropathic pain is not solely a spinal phenomenon, but arises from widespread cortical and subcortical reorganization. However, some studies have reported that cortical reorganization may be limited or differentially expressed in individuals with pain, indicating that the relationship between plasticity and neuropathic pain remains complex, and potentially involves both maladaptive and compensatory mechanisms [38].

Multimodal neuroimaging and electrophysiological evidence have suggested that cortical reorganization following SCI comprises both spatial and functional changes that develop progressively over time. One study by Lotze et al. showed that combined fMRI–TMS findings demonstrate that motor representations shift toward deafferented cortical regions, accompanied by region-specific alterations in cortical excitability, with both processes correlating with injury duration [20]. EEG studies further revealed persistent long-term neuroplasticity, characterized by reduced motor-related potentials during attempted movement, but partial preservation of preparatory neural activity, indicating maintained central motor programming, despite paralysis [22]. Longitudinal fMRI data also showed that cortical reorganization is dynamic and functionally relevant, with early compensatory recruitment of widespread sensorimotor areas gradually normalizing towards primary motor cortex activation as motor recovery improves [34]. Together, these findings highlight the fact that spatial reorganization and excitability changes occur in parallel, and reflect both the adaptive and compensatory mechanisms underlying neuroplasticity after SCI.

In addition, some studies have suggested that activity-dependent plasticity plays a central role in cortical reorganization following SCI, with paired associative stimulation (PAS) serving as a key experimental model for probing long-term potentiation (LTP)-like mechanisms in the primary motor cortex. PAS studies have further demonstrated that the ability to induce synaptic plasticity is preserved in individuals with good functional recovery, but is significantly impaired in those with poor outcomes, indicating that disrupted LTP-like processes may underlie maladaptive neuroplasticity [17,21,44]. These findings support the concept that recovery after SCI depends not only on structural or spatial reorganization but also on the capacity of residual neural circuits to undergo activity-dependent strengthening. Consequently, the PAS and related neuromodulatory approaches provide tools to assess cortical plasticity and potential therapeutic strategies to enhance adaptive motor recovery.

### 4.3. Propriospinal Reorganization

Six studies reported propriospinal reorganization in patients with SCI. Neuroimaging studies have reported evidence of spinal, while propriospinal reorganization was reported in one neuroimaging study [41]. Structural and functional MRI analyses demonstrated that motor recovery 6 months after SCI was associated with preserved spinal cord integrity and adaptive changes in sensorimotor networks. Individuals with greater functional recovery show more organized activation patterns and better structural preservation, indicating that reorganization within the spinal and supraspinal pathways may contribute to motor improvement after SCI [41].

Neuromodulation-based studies have additionally suggested that residual spinal pathways are capable of activity-dependent plasticity. Paired stimulation protocols targeting the motor cortex and spinal cord designed to induce Hebbian plasticity are associated with improvements in voluntary motor output and corticospinal transmission in individuals with chronic SCI [52], indicating that synchronized stimulation may strengthen spared neural pathways and promote functional reorganization within descending and spinal circuits.

Functional imaging studies of the spinal cord have also provided evidence of intrinsic spinal network plasticity after SCI [33]. fMRI of the spinal cord demonstrated task-related neural activity in the spinal segments above and below the lesion level, indicating preserved functional responsiveness, despite structural disruption [33]. Altered activation patterns further suggest the reorganization of segmental networks and residual spinal pathways, supporting the presence of adaptive plasticity within the spinal circuitry following injury.

The conceptual framework of SCI recovery highlights the role of neural plasticity across multiple levels of the nervous system. Evidence from both experimental and clinical studies has indicated that recovery may involve compensatory motor strategies, activity-dependent reorganization of spared neural circuits, and limited structural repair, such as axonal sprouting and synaptic remodeling [23]. Collectively, these mechanisms contribute to functional adaptation after SCI, and may be enhanced by rehabilitation interventions.

Direct neurophysiological evidence of propriospinal pathway involvement has been reported in studies examining interlimb reflexes in individuals with chronic SCI. Further, reflex-based stimulation paradigms have demonstrated that the afferent input from one limb can trigger sustained motor activity in muscles below the lesion level [35]. These responses suggest the presence of functional ascending propriospinal connections capable of transmitting signals between spinal segments, despite disrupted supraspinal control.

Finally, neuromodulation studies targeting spinal inhibitory circuits have indicated that spinal inter-neuronal networks remain modifiable after injury. Combined anodal transcranial direct current stimulation and patterned peripheral electrical stimulation have been shown to influence the spinal inhibitory interneurons involved in reflex modulation and motor control [40]. These findings suggest that cortical and peripheral stimulation can modulate spinal network excitability, supporting the potential of therapeutic interventions to facilitate adaptive spinal plasticity after SCI.

### 4.4. Neurochemical and Molecular Changes

Neurochemical and molecular adaptations were also reported as important components of neuroplasticity following SCI in two studies. One study highlighted the role of neurotrophic factors in promoting neuronal survival, axonal sprouting, and synaptic remodeling after injury [16]. Experimental evidence has further indicated that molecules such as brain-derived neurotrophic factor (BDNF), nerve growth factor (NGF), and neurotrophin-3 (NT-3) can enhance neuronal regeneration and support the reorganization of corticospinal and sensory pathways [16]. These molecular mediators facilitate activity-dependent plasticity by modulating synaptic transmission and promoting structural remodeling within injured neural networks.

In addition to neurotrophins, molecular signaling pathways associated with axonal growth and synaptic plasticity also influence functional recovery after SCI. Studies examining corticospinal tract responsiveness have additionally reported that specific molecular signaling profiles may predict the growth state of corticospinal axons and the degree of neuromodulation-induced muscle response plasticity [20]. Individuals exhibiting molecular markers consistent with a pro-growth state demonstrated greater responsiveness to neuromodulatory interventions, suggesting that intrinsic biological factors contribute to the variability in neural plasticity and rehabilitation outcomes.

Together, these findings indicate that neuroplasticity after SCI is supported not only by the structural and functional reorganization of neural circuits, but also by the molecular mechanisms regulating neuronal survival, axonal growth, and synaptic remodeling. Such neurochemical processes may influence the capacity of the injured nervous system to respond to rehabilitation or neuromodulation therapies, and therefore represent potential therapeutic targets for enhancing recovery after SCI.

### 4.5. Axonal Regeneration

Axonal regeneration following SCI can be distinguished from axonal sprouting. Regeneration refers to new growth arising from an injured axonal stump at or near the lesion, whereas sprouting arises from spared axons adjacent to or remote from the lesion. This distinction is important, as both processes contribute to post-injury plasticity; however, true regeneration is particularly constrained in the adult central nervous system (CNS) by both neuronal intrinsic and environment-dependent mechanisms [56].

One central concept emerging from the neurotrophin literature is that failed regeneration following SCI reflects the interaction between an intrinsically low-growth neuronal state and an extrinsically inhibitory lesion milieu [56]. Intrinsic barriers include insufficient expression or axonal localization of growth-promoting receptors, attenuated activation of pro-growth signaling cascades, and reduced expression of regeneration-associated genes, such as GAP-43 [56]. Extrinsic barriers include the lack of a permissive growth substrate, reactive astrogliosis, inhibitory extracellular matrix molecules, myelin-associated inhibitors, inflammatory changes, repulsive guidance cues, and diminished trophic support [56]. Accordingly, successful regeneration requires a combined strategy that simultaneously enhances neuronal growth competence and remodels the injured microenvironment into a more permissive state [56].

Neurotrophins are particularly relevant to this process as they act at several levels of regenerative biology, supporting neuronal survival, reverse axotomy-induced atrophy, activate growth-associated transcriptional programs, and promoting the elongation of injured axons. In experimental SCI models, NGF, BDNF, and NT-3 have partially distinct trophic specificities, recruiting different axonal populations for growth responses [56]. Among these, NT-3 is particularly important for the ascending sensory pathways, including dorsal column afferents, whereas BDNF predominantly affects the rubrospinal, raphaespinal, coerulospinal, local motor, and proprioceptive sensory axons. Importantly, neurotrophin action is not merely trophic in terms of survival; it is also instructive, particularly when delivered as a gradient that can provide chemotropic guidance to regenerate axons across the lesion and back into the host tissue [56].

A key mechanistic insight is that regeneration depends not only on factor availability, but also on the growth state of the injured neurons. Sensory neurons provide the clearest example; following a peripheral conditioning lesion, dorsal root ganglion neurons upregulate a broad regeneration-associated transcriptional program, including increased BDNF, elevated cAMP/PKA signaling, and enhanced GAP-43 expression [56]. In this primed state, chronically injured sensory axons regenerate into and beyond the permissive grafts when combined with NT-3 delivery. As such, axonal regeneration after SCI is best understood as a state-dependent process in which trophic signals become effective only when intrinsic regenerative competence is sufficiently reactivated [56].

In our opinion, the human study by Calancie et al. did not directly demonstrate axonal regeneration at the lesion site, but did provide clinically important evidence consistent with regenerative sprouting/new intraspinal synaptic growth below the lesion [16]. In individuals with chronic, motor-complete cervical SCI, lower-limb stimulation elicits short-latency motor responses in the distal upper-limb muscles, a phenomenon absent in motor-incomplete SCI and able-bodied subjects [16]. These interlimb reflexes were interpreted as evidence that ascending lower limb afferents deprived of their usual supralesional targets following severe SCI may develop novel synaptic contacts with partially denervated cervical motoneurons caudal to the lesion. The delayed emergence of these reflexes over months, their broad multimodal receptive fields, and their electrophysiological properties all suggest post-injury structural reorganization rather than simple unmasking of latent pathways [16].

Taken together, these studies support a broader mechanistic view in which axonal regeneration following SCI is not a unitary process, but rather a continuum of structural plasticity involving the reactivation of intrinsic growth programs in injured neurons, provision of trophic and guidance cues, particularly neurotrophin modification of the inhibitory lesion milieu through permissive substrates or grafts, and formation of new synaptic relays or intraspinal connections that may persist or emerge in the chronic stage. Neurotrophin-based experimental studies have primarily highlighted lesion-bridging axonal regeneration in animal models, whereas human electrophysiological studies strongly suggest post-lesional intraspinal rewiring or regenerative sprouting, rather than long-distance anatomical regeneration. Accordingly, in the context of neuroplasticity following SCI, axonal regeneration should be conceptualized as a spectrum extending from the true regrowth of injured axons across the lesion to the structurally mediated reorganization of the caudal spinal circuitry, both of which can underlie either adaptive functional recovery or maladaptive plasticity.

### 4.6. Synaptic Plasticity

Synaptic plasticity is an important component of neuroplasticity following SCI, which occurs through the strengthening and reorganization of synaptic connections at both the spinal and supraspinal levels. Electrophysiological evidence from Calancie et al. has demonstrated that interlimb reflexes emerging after cervical SCI progressively increase in strength over time, suggesting the formation and potentiation of novel synaptic connections between afferent inputs and motoneurons [19]. Similarly, Shields et al. reported altered H-reflex depression following SCI, with repetitive muscle activation partially restoring presynaptic inhibition, indicating activity-dependent synaptic reorganization within the spinal circuits [32]. Persistent segmental synaptic plasticity is further supported by vibration-training studies showing recovery from paired-pulse H-reflex depression, even in chronic SCI [55]. Changes in peripheral motor axon excitability after cervical SCI also indicate trans-synaptic adaptations that influence motor neuron input during recovery [27]. Neuroimaging findings complement these results; for example, Hou et al. showed that improved motor recovery was associated with increased functional connectivity among the primary motor and higher-order motor areas, reflecting strengthened synaptic interactions within the corticospinal networks [41]. Large-scale connectivity analysis further demonstrated increased sensorimotor and cerebellar network connectivity after SCI, indicating compensatory synaptic strengthening across distributed brain networks [42]. In addition, neuromodulation studies have reported that transcutaneous spinal stimulation alters intracortical inhibition and enhances motor-evoked responses, consistent with bidirectional synaptic plasticity at the cortical and spinal levels [46]. Structural and functional changes in the extrapyramidal circuits are also associated with recovery, suggesting synaptic reorganization within the basal ganglia–thalamic pathways [47]. Collectively, these findings indicate that synaptic plasticity after SCI involves the strengthening of residual pathways, formation of new spinal synapses, and modulation of inhibitory circuits, contributing to functional adaptation following injury.

### 4.7. Maladaptive Plasticity

In addition to adaptations that promote recovery, the CNS also develops mechanisms of undergoing functional and structural reorganization, characterized by “maladaptive plasticity.” Owing to the disruption of afferent and efferent pathways following injury, the brain cortex and spinal cord regions deprived of input automatically establish abnormal connections or remove natural inhibitory controls [14]. Consequently, patients commonly experience persistent and debilitating complications, such as central neuropathic pain, spasticity, and hyperreflexia [14].

In patients with SCI, deafferented cortical areas tend to be occupied by representative regions of adjacent intact body parts. Evidence from fMRI has indicated that in patients with SCI experiencing neuropathic pain below the level of injury, the activation site for the little finger on the S1 cortex shifts medially, encroaching on the cortical region previously responsible for the legs [26]. Notably, this cortical shift correlated positively with the severity and intensity of neuropathic pain experienced by patients. This reorganization is considered a “misadaptive” response that creates a pathological loop, in which normal tactile signals from the hand are amplified into the phantom pain impulses that cascade down to the spinal cord below the injury, maintaining a state of intractable central pain [26].

In cases of motor function impairment, during the subacute phase following SCI, fMRI reveals reduced activation of the primary motor cortex, whereas the associated sensorimotor regions show hyperactivation. Cortical regions that previously controlled paralyzed body parts now participate in activating movement in unaffected body parts. At the brainstem and spinal cord levels, the loss of descending inhibitory signals leads to exaggeration of brainstem reflexes and spontaneous firing of motor neurons, even at rest [22].

### 4.8. Other Mechanisms

Beyond the commonly described mechanisms such as cortical reorganization and synaptic plasticity, a small number of studies have highlighted other neuroplastic mechanisms in SCI, particularly involving brainstem circuitry and neurotransmitter-mediated modulation. For example, Kumru et al. demonstrated that SCI is associated with increased excitability and reduced inhibition of brainstem inter-neuronal circuits, as evidenced by enhanced blink reflex recovery and impaired pre-pulse inhibition [29]. These findings suggest a disruption of inhibitory control, likely related to reduced GABAergic activity, which could contribute to abnormal sensorimotor integration after injury. Importantly, administration of intrathecal baclofen restored these abnormalities toward normal levels, emphasizing the role of neurotransmitter-dependent plasticity in modulating neural circuits after SCI [29].

In addition, Versace et al. showed that LTP-like synaptic mechanisms at the cortical level are not uniformly preserved after SCI, particularly in patients with poor functional recovery [44]. Using PAS, they found reduced ability to induce corticospinal excitability changes, suggesting impaired activity-dependent associative plasticity [44]. This highlights that beyond structural or network reorganization, SCI also involves alterations in the capacity for inducing plastic changes, which may directly relate to functional outcomes.

Together, these studies indicate that “other mechanisms” of neuroplasticity in SCI include neurotransmitter imbalance, altered brainstem circuit regulation, and impaired plasticity induction capacity, which extend beyond traditional cortical and spinal reorganization models may play a critical role in both recovery and maladaptation.

Overall, this review identified several knowledge gaps. First, most existing studies have focused on cortical changes, whereas spinal and molecular mechanisms have been investigated less frequently. Second, heterogeneity in the study design, injury characteristics, and outcome measures limits comparisons across studies. Third, longitudinal data examining the temporal evolution of neuroplasticity from acute to chronic phases remain limited. Fourth, the relationship between adaptive plasticity, functional recovery, and maladaptive plasticity remains unclear. Finally, few studies have integrated multimodal approaches combining structural, functional, electrophysiological, and clinical outcomes, which are necessary to better characterize the neuroplastic mechanisms after SCI.

Despite the growing number of studies investigating neuroplasticity following SCI, the current evidence remains limited by several methodological challenges. Many included studies were cross-sectional, pilot, case-series, or case-report designs with relatively small sample sizes, limiting statistical power and generalizability. In addition, substantial heterogeneity existed across the SCI populations regarding injury level, completeness, chronicity, functional status, and rehabilitation exposure. Variability in neuroimaging and electrophysiological methodologies further complicates direct comparison across studies and may partly explain inconsistent findings regarding adaptive and maladaptive plasticity. Therefore, current evidence should be interpreted cautiously, and future research should prioritize larger longitudinal studies with standardized outcome measures and better participant stratification to clarify the mechanisms and clinical relevance of neuroplasticity following SCI.

Future studies should adopt multimodal and longitudinal designs to better characterize the neuroplastic changes across different stages of spinal cord injury. The combination of neuroimaging, electrophysiological assessments, and clinical outcomes may provide a more comprehensive understanding of both adaptive and maladaptive plasticity. Greater emphasis should additionally be placed on investigating spinal and propriospinal mechanisms as well as neurochemical and molecular pathways that may influence recovery.

Standardization of outcome measures and neuroplasticity definitions is recommended to improve comparability across studies. In addition, future research should explore the interaction between rehabilitation interventions and neuroplastic mechanisms, particularly the activity-dependent plasticity induced by task-specific training, neuromodulation, and combined therapeutic approaches. Identifying biomarkers of adaptive versus maladaptive plasticity can also help to guide individualized rehabilitation strategies.

Finally, larger prospective studies are needed to clarify the relationship between neuroplastic changes and functional recovery as well as to determine the optimal timing and intensity of rehabilitation interventions aimed at promoting beneficial neuroplasticity while minimizing maladaptive outcomes.

## 5. Conclusions

Neuroplasticity following spinal cord injury involves multiple interacting mechanisms at the cortical, subcortical, and spinal levels. Cortical reorganization and activity-dependent plasticity are the most commonly reported processes, whereas propriospinal reorganization, synaptic plasticity, and neurodegenerative changes contribute to functional adaptation. However, axonal regeneration and its neurochemical mechanisms remain poorly characterized in human studies. Importantly, neuroplasticity following SCI may be both adaptive and maladaptive, influencing functional recovery and complications such as neuropathic pain. A better understanding of these mechanisms may help to guide rehabilitation strategies aimed at enhancing beneficial plasticity and improving the outcomes in individuals with spinal cord injury.

## Figures and Tables

**Table 1:** Characteristics of included studies.

Studies	Study Design	Sample Size *n* (SCI/ Healthy)	Level of Injury	Severity	Duration	Methodological Approach	Axonal Regeneration	Synaptic Plasticity	Cortical Reorganization	Propriospinal Pathway Reorganization	Neurochemical and Molecular Changes	Activity-Dependent Plasticity	Maladaptive Plasticity	Other Mechanisms
Calancie B. (1996) [16]	Cross-sectional experimental study	15 SCI	Cervical	Motor complete	>1 year	EMG	x				x			
Green J.B. (1998) [17]	Cross-sectional case–control study	23 (12/11)	C2–T12	Complete and incomplete	9–192 month	High-resolution EEG, EMG			x					
Curt A. (2002) [18]	Cross-sectional case–control study	22 (14/8)	C5–L2	Complete	Chronic	PET			x					
Calancie B. (2005) [19]	Longitudinal observational study	40 SCI	Cervical	Complete and incomplete	Subacute and chronic	EMG		x						
Lotze M. (2006) [20]	Cross-sectional case–control study	12 (6/6)	T3–L1	Complete and incomplete	Subacute and chronic	fMRI, TMS, EMG		x	x		x	x		
Hoffman L.R. (2007) [21]	Case report	1	C6	Incomplete	1 year	TMS			x			x	x	
Jurkiewicz M.T. (2007) [22]	Case series	16 (6/10)	C5-C8	NA	Subacute	fMRI			x			x	x	
Curt A. (2008) [23]	Prospective multicenter longitudinal cohort study	460 SCI	Tetraplegia and paraplegia	Complete and incomplete	Acute	TMS, EMG				x		x		
Enzinger C. (2008) [24]	Case report with control comparison	6 (1/5)	C4	Complete	Chronic	fMRI, EEG						x		
Wrigley P.J. (2009) [25]	Cross-sectional case–control	42 (15/27)	T1–T10	Complete	24–390 months	MRI		x	x					
Wrigley P.J. (2009) [26]	Cross-sectional case–control study	41 (20/21)	T1–T10	Complete	2–37 years	fMRI			x				x	
Boland R.A. (2009) [27]	Case report	1 SCI	C6	Incomplete	Acute	EMG		x				x		
Duggal N. (2010) [28]	Prospective longitudinal cohort study	22 (12/10)	Cervical	Incomplete	Preoperative and 6 months post-surgery	fMRI		x	x				x	
Kumru H. (2010) [29]	Cross-sectional case–control study	41 (28/13)	C4–T12	Complete and incomplete	Chronic	EMG								x
Gustin S.M. (2010) [30]	Cross-sectional comparative study	68 (23/45)	T2–T10	Complete	13–16 years	MRI			x					
Henderson L.A. (2011) [31]	Cross-sectional case–control study	43 (20/23)	T1–T10	Complete	2–37 years	fMRI			x					
Shields R.K. (2011) [32]	Prospective longitudinal interventional study	15 SCI	C5–T10	NA	Acute and subacute	EMG		x				x		
Cadotte D.W. (2012) [33]	Cross-sectional case–control study	38 (18/20)	C2-C7	Complete and incomplete	Chronic	fMRI		x		x				
Castro A. (2013) [34]	Cross-sectional case–control study	29 (9/20)	C4–T10	Complete	6–24 years	EEG			x			x		
McNulty P.A. (2013) [35]	Case report	1 SCI	C5	Complete	20 years	EMG, TMS				x				
Di Rienzo F. (2014) [36]	Single-arm longitudinal interventional study	12 (6/6)	C6-C7	Motor complete	>6 months	MEG			x			x		
Di Rienzo F. (2014) [37]	Pilot longitudinal interventional study	8 (4/4)	C6	Complete	6–32 months	MEG						x		
Jutzeler C.R. (2015) [38]	Cross-sectional case–control study	55 (24/31)	C6–L3	Complete and incomplete	4–33 years	fMRI			x					
Oni-Orisan A. (2016) [39]	Prospective case–control study	20 (11/9)	C4–C7	Complete	>2 years	fMRI			x					
Yamaguchi T. (2016) [40]	Randomized crossover trial	21 (11/10)	C4–T11	Incomplete	0.6–12 years	tDCS, EMG				x				
Hou J. (2016) [41]	Prospective observational study	50 (25/25)	C5–T12	Complete and incomplete	2–12 weeks	DTI, fMRI			x	x				
Kaushal M. (2017) [42]	Cross-sectional case–control study	30 (15/15)	C4–C7	Complete	3–36 years	fMRI		x	x					
Urbin M.A. (2017) [43]	Non-randomized interventional study	41 (18/23)	C2–T11	Complete and incomplete	>1 year	TMS, EMG		x						
Versace V. (2018) [44]	Pilot study	20 (10/10)	C5–C7	Incomplete	4–17 years	TMS, EMG		x	x					
Bunday K.L. (2018) [45]	Randomized crossover study	31 (17/14)	C3–C8	Complete and incomplete	1–20 years	TMS, EMG						x		
Benavides F.D. (2020) [46]	Randomized crossover trial	32 (17/15)	C4–C6	Complete and incomplete	Chronic	TMS, EMG, MRI								x
Huber E. (2020) [47]	Prospective cohort study	39 (17/21)	C3–T12)	Complete and incomplete	Subacute	MRI			x					
Jo H.J. (2020) [48]	Randomized controlled trial	38 SCI	C2–L3	Complete and incomplete	>1 year	TMS, EMG		x				x		
Zhang Z. (2021) [49]	Pilot case series	3 SCI	C4–C5	Incomplete	1–6 years	TMS, EMG						x		
Nakanishi T. (2021) [50]	Cross-sectional study	18 (8/10)	T10–L1	Motor complete	Chronic	fMRI			x					
Zaaya M. (2021) [51]	Pilot case series	4 SCI	C6–T9	Motor complete	Chronic	EMG						x		
Jo H.J. (2023) [52]	Randomized controlled trial	31 SCI	C2–T10	Complete and incomplete	>1 year	TMS, EMG		x		x				
Summary							1	12	19	6	2	14	4	2

DTI, Diffusion Tensor Imaging; EEG, Electroencephalography; EMG, Electromyography; fMRI, Functional Magnetic Resonance Imaging; MEG, Magnetoencephalography; MRI, Magnetic Resonance Imaging; NA, Not Available; PET, Positron Emission Tomography; SCI, Spinal Cord Injury; tDCS, Transcranial Direct Current Stimulation; TMS, Transcranial Magnetic Stimulation.

## Data Availability

The original contributions presented in this study are included in the article and Supplementary Materials. Further inquiries can be directed to the corresponding authors.

## Supplementary Materials

The following supporting information can be downloaded at: https://www.mdpi.com/article/10.3390/biomedicines14091963/s1, Table S1: PRISMA 2020 for Abstracts Checklist; Table S2: Preferred Reporting Items for Systematic reviews and Meta-Analyses extension for Scoping Reviews (PRISMA-ScR) Checklist.

## Appendix A. PUBMED Search Strategy

#1 Spinal Injuries [MeSH Terms] OR Spinal Cord Injuries [MeSH Terms] OR Central Cord Syndrome [MeSH Terms] OR Spinal cord ischemia [MeSH Terms]

#2 Cord injury” [Title/Abstract] OR “Central cord syndrome” [Title/Abstract] OR “quadriplegi *” [Title/Abstract] OR “tetraplegi *” [Title/Abstract] OR “spinal cord injury” [Title/Abstract] OR “spinal injury” [Title/Abstract] OR “spinal cord ischemia” [Title/Abstract] OR “SCI” [Title/Abstract]

#3 #1 or #2

#4 Neuronal Plasticity [MeSH Terms] OR Cell Plasticity [MeSH Terms]

#5 Neuroplasticity [Title/Abstract] OR “axon regeneration” [Title/Abstract] OR “Neuron regeneration” [Title/Abstract] OR “Neural reorganization” [Title/Abstract] OR “neurogenesis” [Title/Abstract] OR “Plasticity” [Title/Abstract] OR “Neural plasticity” [Title/Abstract] OR “brain plasticity” [Title/Abstract] OR “synaptic plasticity” [Title/Abstract] OR “spinal plasticity” [Title/Abstract] OR “cortical plasticity” [Title/Abstract].

#6 #4 OR #5

#7 #3 AND #6

## Appendix B. SCOPUS Search Strategy

TITLE-ABS-KEY ((SCI OR {Cord injury} OR {Central cord syndrome} OR quadriplegi! OR tetraplegi! OR {spinal injury} OR {spinal cord ischemia}) AND (Neuroplasticity OR {axon regeneration} OR {Neural reorganization} OR neurogenesis OR {plasticity} OR {Neural plasticity} OR {brain plasticity} OR {synaptic plasticity} OR {spinal plasticity} OR {cortical plasticity}))

## Appendix C. CENTRAL Search Strategy (Cochrane Library)

#1 MeSH descriptor: [Spinal Cord Injuries] explode all trees

#2 MeSH descriptor: [Spinal Cord Ischemia] explode all trees

#3 MeSH descriptor: [Spinal Injuries] explode all trees

#4 MeSH descriptor: [Quadriplegia] explode all trees

#5 MeSH descriptor: [Central Cord Syndrome] explode all trees

#6 (“Cord injury” OR “Central cord syndrome” OR “quadriplegi *” OR “tetraplegi *” OR “central cord syndrome” OR “spinal cord injury” OR “spinal injury” OR “spinal cord ischemia” OR “SCI”): ti, ab, kw

#7 #1 or #2 or #3 or #4 or #5 or #6

#8 MeSH descriptor: [Neuronal Plasticity] explode all trees

#9 (Neuroplasticity or “axon regeneration” or “Neuron regeneration” OR “Neural reorganization” OR “neurogenesis” OR “Plasticity” OR “Neural plasticity” OR “brain plasticity” OR “synaptic plasticity” OR “spinal plasticity” OR “cortical plasticity”): ti, ab, kw

#10 #8 OR #9

#11 #7 AND #10
